# Immune-epithelial crosstalk governs immune checkpoint inhibitor efficacy in colorectal cancer

**DOI:** 10.1016/j.isci.2026.116544

**Published:** 2026-07-08

**Authors:** Sheah Lin Lee, Adrian C. Bateman, Margaret Ashton-Key, John N. Primrose, Alex Mirnezami, Aymen Al-Shamkhani, Stephen M. Thirdborough

**Affiliations:** 1 School of Cancer Sciences, University of Southampton, Tremona Road, Southampton, UK; 2 University Hospital Southampton NHS Foundation Trust, Tremona Road, Southampton, UK; 3 Antibody and Vaccine Group, Faculty of Medicine, School of Cancer Sciences, Centre for Cancer Immunology, University of Southampton, Southampton, UK

**Keywords:** mismatch repair-deficient colorectal cancer, T and B cell clonality, checkpoint inhibitor, immune-epithelial crosstalk, spatial transcriptomics

## Abstract

Colorectal cancer (CRC) with deficient DNA mismatch repair (dMMR) elicits a CD8^+^ T cell response. However, the magnitude and composition of this response vary among patients, influencing the efficacy of immune checkpoint inhibitors (ICIs). To investigate this heterogeneity, we integrated bulk transcriptomics with immune repertoire sequencing in dMMR CRC. We identified co-regulated gene modules associated with T- and B cell clonal expansion, immune-metabolic interactions, and therapeutic response. Key transcriptional programs linked to T cell clonality and ICI responsiveness were validated using spatial transcriptomics and the TCGA-CRC cohort. We observed an inverse relationship between immune activation and oxidative metabolism and identified immune-epithelial crosstalk as a determinant of ICI efficacy. These findings may inform biomarker development and patient stratification in both dMMR and mismatch repair-proficient CRC. Transcriptional programs linked to T cell clonality in dMMR CRC also define a subset of immunologically active microsatellite-stable tumors, supporting the rationale for extending ICI-based therapies to these patients.

## Highlights

• Immune-epithelial transcriptional modules predict ICI response in dMMR and MSS CRC
• T- and B cell clonal expansion shapes the TME in dMMR CRC
• Spatial transcriptomics links epithelial remodeling to immune activation in CRC

## Graphical abstract

**Figure:**
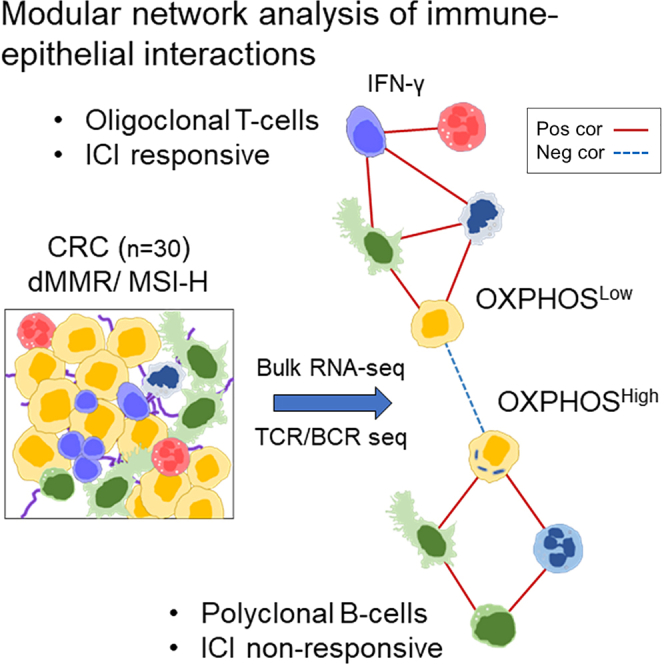

## Teaser

Immunology; Oncology; Biological sciences

## Introduction

Colorectal cancer (CRC) remains a leading cause of cancer-related mortality worldwide, with a 5-year survival rate of only 8%–23% in metastatic cases.^1^ CRC is a heterogeneous disease, shaped by diverse genetic and epigenetic alterations that influence prognosis and therapeutic response.^2^ Approximately 15% of CRCs exhibit deficient DNA mismatch repair (dMMR), with the majority (∼12%) arising sporadically due to MLH1 promoter hypermethylation.^3^^,^^4^ The remainder are hereditary, caused by germline mutations in mismatch repair genes such as MLH1, PMS2, MSH2, or MSH6.^5^^,^^6^ Both sporadic and hereditary dMMR tumors are characterized by microsatellite instability (MSI) and a high tumor mutational burden (TMB), leading to increased neoantigen production.^7^ This immunogenicity drives a robust tumor microenvironment (TME), enriched in CD8^+^ cytotoxic T cells and often accompanied by elevated expression of immune checkpoint molecules.^8^ Consequently, immune checkpoint inhibitors (ICIs) have demonstrated significant clinical benefit in dMMR CRC and are now recommended as first-line therapy for metastatic cases.^9^

Recent trials have shown that early-stage, non-metastatic dMMR CRC also responds well to ICI. For example, the NICHE trial reported a 100% pathological response rate to neoadjuvant dual CTLA-4 and PD-1 blockade in stage I-III dMMR CRC, with 60% achieving a complete pathological response within 6 weeks.^10^ The follow-up NICHE2 trial confirmed these findings, with 98% (109/111) of dMMR patients showing a pathological response and 68% (75/111) achieving a complete pathological response to CTLA-4 and PD-1.^11^ Similarly, a phase 2 trial in stage II-III dMMR rectal cancer reported complete clinical responses in all patients treated with PD-1 blockade.^12^ However, this response is maintained in only 49% (20/41) of patients, with a median follow-up of 29 months from the first treatment,^13^ indicating heterogeneity within dMMR CRC.

Proficient mismatch repair (pMMR) CRC, including microsatellite stable (MSS) and MSI-low (MSI-L) tumors^14^ is typically “immune cold”, with limited immune infiltration and poor responsiveness to ICI.^15^ For instance, the objective response rate to PD-1 or combined PD-1/CTLA-4 blockade in pretreated metastatic MSS CRC is <1%.^15^^,^^16^ Stratification by TMB (≥9 mutations/Mb) in the TAPUR trial showed minimal benefit, leading to early termination.^17^ However, recent advances suggest that a subset of MSS tumors may still benefit from ICI. In a phase I/II trial, the combination of PD-1 blockade with an Fc-enhanced anti-CTLA-4 antibody (botensilimab^18^) achieved a 17% response rate in metastatic MSS CRC, including one complete response among 101 patients.^19^ Similarly, in a small cohort of 19 pMMR CRC, neoadjuvant treatment with PD-1 blockade (nivolumab) induced a major pathological response in three patients.^20^ Since the pMMR subset, which constitutes 85% of CRC cases, is currently ineligible for ICI treatment, identifying biomarkers beyond MMR status or TMB is urgently needed.

This study aims to characterize the heterogeneity of early-stage dMMR CRC and identify transcriptional programs relevant to pMMR tumors. We used a network-based approach to define gene co-expression modules from bulk RNA sequencing (bulk RNA-seq) data. These modules were integrated with T- and B cell receptor repertoire sequencing to characterize immune and metabolic features of the TME. Our analysis revealed modules associated with T- and B cell clonal expansion, immune-metabolic interactions, and potential responsiveness to ICI. Spatial transcriptomics further validated key immune-epithelial interactions. These findings offer insights into the immune architecture of dMMR CRC and suggest that clonally expanded T cell programs in MSI-high (MSI-H) tumors may also be present in a subset of MSS tumors, offering a potential biomarker framework for identifying ICI-responsive “hot” MSS CRC.

## Results

### Study cohort

We collected tumor and adjacent normal mucosal tissue from thirty treatment-naïve patients (SOTON cohort) for bulk and immune repertoire sequencing. The clinical and histopathological classification of these patients is summarized in Table S1. The median age of the cohort was 77 years (range 53–89). There was a predilection for right-sided cancer (*n* = 29) and for female patients (*n* = 20), which are recognized characteristics of dMMR CRC^21^⁠. Consistent with previous work,^5^⁠ the majority of dMMR CRC had loss of MLH1 and PMS2 protein expression (*n* = 23), while the remaining patients displayed a mixed pattern of loss (Table S1).

### Immune and metabolic pathway diversity in dMMR CRC

To uncover co-regulated gene programs that define the TME in dMMR CRC, we first performed a weighted gene co-expression network analysis (WGCNA). This systems-level approach identifies gene modules that reflect underlying biological processes and cellular states.

**Figure 1:**
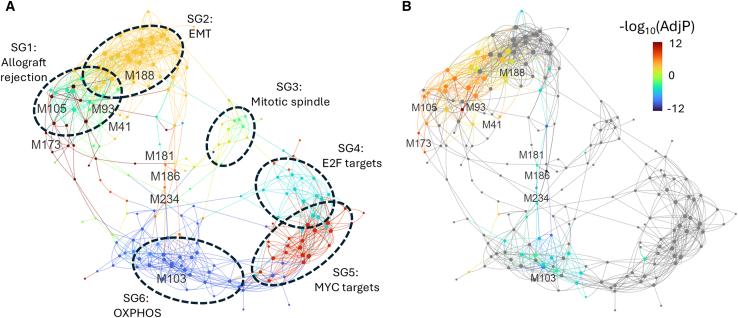
Module eigengene network feature analysis Spring-embedded layouts with edges weighted by a correlation coefficient between module eigengenes (MEs) (edge threshold = ρ > 0.87) and node size proportional to the number of edges. (A) Modules are colored according to the results of pre-ranked gene set enrichment analysis (GSEA), with genes ranked using a module membership (kME) metric. Network subgraphs are highlighted and labeled with their top associated MSigDB hallmark pathway. Selected modules are also labeled with their numerical assignment. (B) Modules are colored using a signed log10-transformed Adj*p* value from differential analysis (MSI-H versus MSS), with the sign indicating the direction of change.

The gene co-expression network, comprising 14,916 actively expressed genes, was partitioned into 239 modules (for annotations, see Table 1 for selected modules and Table S2 for all 239). Initially, relationships among the modules were examined by constructing an eigengene network (EGN), in which each module’s gene expression matrix is summarized by its first principal component, and edges reflect the connection strength (Spearman rho) between these module eigengenes (MEs).^22^ The EGN exhibits six main subgraphs (SG) of connected modules, each enriched for distinct biological processes (Figure 1A), including the TME-related signatures “Allograft rejection” (SG1) and “Epithelial-mesenchymal transition” (SG2).

**Table 1:** Key Reactome pathways and statistical analyses for selected modules

Module	modSize	pres Zsummary	Reactome pathway	Adj*p*	CoxPH log rank	CoxPH Tstat
M41	47	1.7	cyclin A-associated events at S-phase entry	2.4e−03	7.5e−02	1.51
M59	75	9.1	interferon αβ-signaling	1.4e−13	1.1e−02	2.58
M93	43	5.5	NOD1-2 signaling pathway	4.8e−03	1.6e−03	3.27
M103	64	11.0	triglyceride metabolism	5.7e−04	8.7e−02	1.70
M105	96	50.9	adaptive immune system	1.1e−12	2.9e−03	2.96
M130	76	4.9	CD22-mediated BCR regulation	2.2e−02	5.3e−02	−2.03
M173	56	28.3	interferon signaling	1.2e−19	4.8e−02	2.15
M181	101	57.1	CD22-mediated BCR regulation	5.5e−92	5.8e−03	2.58
M186	34	10.8	regulation of TP53 expression and degradation	3.2e−03	2.1e−02	2.50
M188	92	41.2	extracellular matrix organization	5.4e−30	5.5e−02	1.94
M229	87	9.2	downregulation of TGF-β receptor signaling	1.0e−02	1.5e−01	1.49
M234	52	9.3	integrin cell surface interactions	3.2e−03	6.9e−03	2.85
M235	106	37.4	eukaryotic translation elongation	2.9e−145	1.0e−01	1.50

This table presents the top Reactome pathways, and their Benjamini-Hochberg (BH) adjusted *p* values (Adj*p*) for selected modules. It also includes log-rank *p* values and T-statistics from univariate Cox proportional hazards regression analysis. Additionally, module preservation scores in TCGA-CRC test data are listed, with values below 2 indicating non-preservation.

To assess the reproducibility of these modules in an independent dataset, we evaluated their preservation in TCGA-CRC cohort, which includes MSI-H (*n* = 37), MSI-L (*n* = 40), and MSS (*n* = 121) tumors.^23^ Of the 239 modules identified in the SOTON cohort, 84.9% were preserved in TCGA-CRC dataset (Figure S1 and Table S2). This high level of preservation underscores the robustness and generalizability of our network structure.

Having confirmed the reproducibility of the module network, we next investigated whether specific modules were associated with MSI status in TCGA-CRC cohort. We identified 59 significant modules (Adj*p* <0.01) that were differentially expressed between MSI-H and MSS tumors (see Figure S2 for the top 10 modules ranked by Dunn’s test with BH adjustment). Modules with higher expression in MSI-H tumors were predominantly located within the immune-related subgraph (SG1, Figure 1B). In contrast, those elevated in MSS/MSI-L tumors were primarily components of the oxidative phosphorylation (OXPHOS) subgraph (SG6, Figure 1B). This differential pattern highlights distinct TME characteristics associated with MSI status.

**Figure 2:**
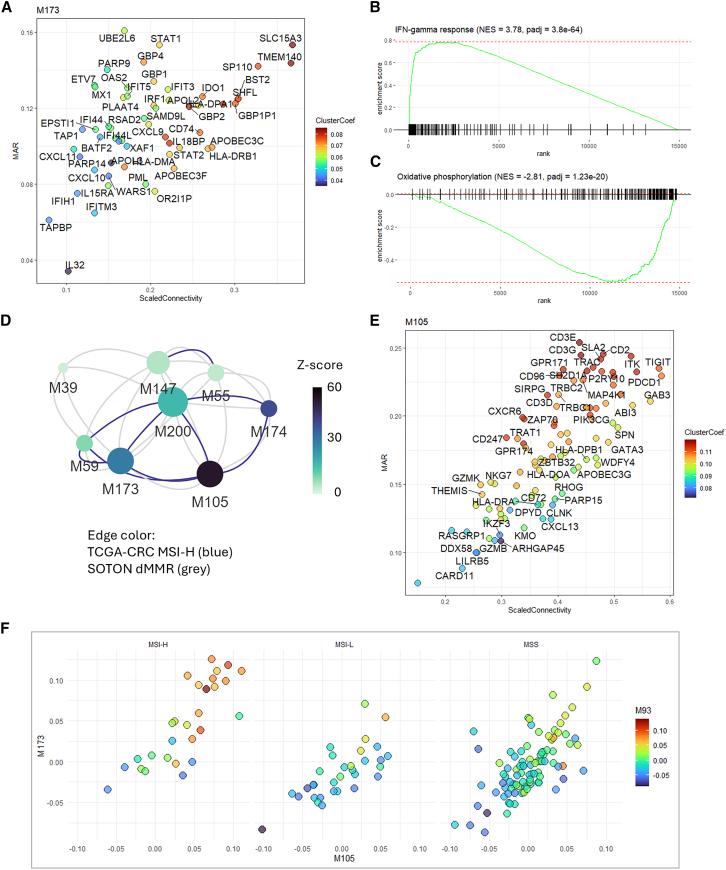
Strong association between IFN-γ responses and CD8 αβ-T cells in dMMR (A) Scatterplot of scaled connectivity versus the maximum adjacency ratio (MAR) for M173. (B and C) GSEA plots for the MSigDB hallmark IFN-γ response and oxidative phosphorylation, with all expressed genes pre-ranked by a module membership metric for M173. (D) Results of the RWR algorithm with M173 as the seed node, showing the top seven k-ranked neighbors. Edge threshold ρ > 0.87. Nodes are colored by module preservation *Z* scores, using the SOTON dMMR expression data as the reference and TCGA-CRC MSI-H as an independent test dataset. (E) Scatterplot of scaled connectivity versus MAR for M105. (F) Scatterplot of M105 (PD1^+^, CD8^+^ T cell) versus M173 (IFN-γ response) module expression, split by MSI status, with points colored by M93 (PDL1^+^, CEACAM4^+^ granulocyte) expression.

Among the highly preserved modules significantly associated with MSI status (Figure S2), M173 was the top-ranked module linked to MSI-H tumors, with a Dunn’s adjusted *p* value of 4.8e−09 (MSI-H vs. MSS). M173 is enriched for interferon (IFN)-related genes, including *CXCL9*, *IRF1*, *PSMB9*, *STAT1*, *STAT2*, and *TAP1* (Figure 2A). To further characterize the internal structure of M173, we applied the module membership metric (kME), which quantifies the correlation between each gene’s expression profile and the module eigengene. A high kME value indicates that a gene is strongly representative of the module’s overall expression behavior. Using this kME-based ranking, we found that the most central genes in M173 were significantly enriched in the MSigDB hallmark pathway “IFN-γ response” (normalized enrichment score [NES] = 3.78, Adj*p* = 3.8e−64; Figure 2B). Conversely, OXPHOS-associated genes were inversely correlated with the M173 eigengene (NES = −2.81, Adj*p* = 1.2e−20; Figure 2C).

To explore the cellular neighborhood of M173 and assess its conservation within TCGA-CRC MSI-H cohort, we calculated the connection strengths (edges) between MEs separately for the SOTON and TCGA mRNA expression data. We then constructed a biplex EGN, representing modules from both datasets as parallel layers, and applied a random walk with restart (RWR) algorithm^24^ to identify conserved inter-module connections. RWR was selected for its ability to capture both local and global network topology, offering a more nuanced view of module conservation across datasets than traditional correlation-based methods. The RWR process computes similarity in multiplex networks by simulating a virtual particle that starts at a seed node and randomly follows network edges, with the potential to jump to its counterpart on a different layer. At each step, there is a restart probability (*p* = 0.7) of returning to the seed, capturing local network connectivity.

Using M173 as the seed node, we identified M59, M105, and M200 as conserved immediate neighbors of M173 (Figure 2D). These modules are significantly enriched for genes associated with type I interferon signaling (Adj*p* = 1.4e−10), CD8 T cells (Adj*p* = 9.7e−68), and macrophages (Adj*p* = 1.2e−07), respectively. The αβ-TCR CD8 T cell module (M105, Figure 2E) includes genes linked to activated effector T cells, such as the T-box transcription factor *TBX21*, *CD96*, lymphocyte activation gene 3 (*LAG3*), programmed cell death protein 1 (*CD274*), *TIGIT*, and components of cytolytic granules (*GZMB*, *GZMH*, *NKG7*, and *PRF1*).

**Figure 3:**
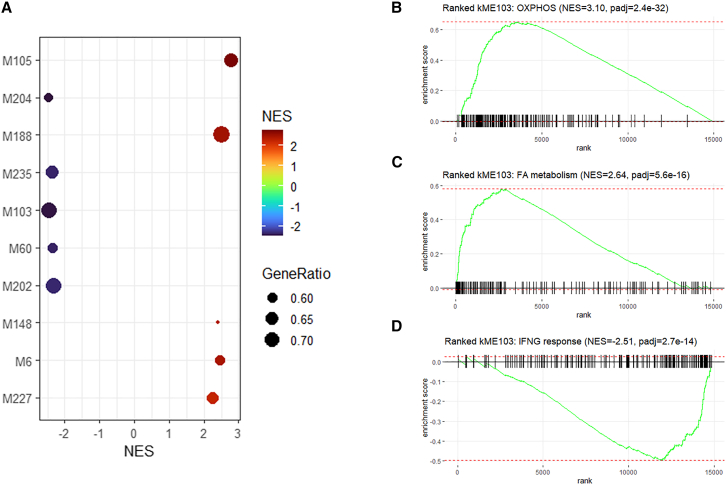
Transcriptional modules associated with ICI response (A) GSEA of SOTON-derived modules in bulk RNA-seq data from MSS CRC patients treated with anti-PD-1 and anti-CTLA-4. (B) GSEA of M103 module genes in TCGA-CRC cohort reveals strong enrichment for OXPHOS pathways. (C) M103 is also enriched for genes involved in fatty acid metabolism, suggesting metabolic reprogramming in MSS tumors. (D) An inverse correlation between M103 expression and IFN-γ response genes highlights a potential immune-metabolic axis of resistance to ICI therapy.

A strong correlation between M173 and M105 expression was observed in MSI-H tumors (ρ = 0.82, Figure 2F), highlighting a robust link between IFN-response pathways and T cell activity in dMMR/MSI-H CRC. This finding aligns with previous reports by Llosa et al.^8^ and, more recently, by Acha-Sagredo et al.^25^ Although the correlation between M173 and M105 was lower in MSS tumors (ρ = 0.64, Figure 2F), approximately 20% of MSS patients exhibited high M173 expression. To assess whether IFN-high immunophenotypes could predict ICI response in MSS CRC, we analyzed bulk RNA-seq data from patients treated with anti-PD-1 and anti-CTLA-4 therapies (9 responders, 18 with stable disease for ≥12 weeks, and 11 with progressive disease).^19^ Gene set enrichment analysis (GSEA) using the SOTON module gene sets revealed that partial responders showed significant enrichment for T cell (M105; NES = 2.82, false discovery rate (FDR) = 7.5e−17) and myofibroblast (M188; NES = 2.57, FDR = 3.5e−11) signatures (Figure 3A). In contrast, progressive disease was associated with elevated expression of modules M103 (NES = 2.49, FDR = 3.3e−11) and M235 (NES = 2.35, FDR = 2.3e−10), the latter of which was significantly enriched for the Reactome pathway “Eukaryotic Translation Elongation” (2.9e−145). M103 includes transcriptional signatures characteristic of differentiated colorectal epithelium (*CDHR5*, *EPCAM*, *GPA33*, and *KRT20*) and serine proteases involved in epithelial barrier function and tumor invasion (*MEP1A*, *PRSS8*, and *ST14*). Additional genes such as *FABP1*, *FMO5*, *MOGAT2*, and *MOGAT3* suggest altered lipid metabolism. A kME-ranked gene list for M103 in TCGA-CRC cohort showed strong enrichment for MSigDB hallmark pathways, ‘Oxidative phosphorylation’ (NES = 3.18, Adj*p* = 3.8e−32; Figure 3B) and “Fatty acid metabolism” (NES = 2.69, Adj*p* = 3.0e−16; Figure 3C), while hallmark “IFN-γ response” genes were inversely correlated with the M103 eigengene (NES = −2.47, Adj*p* = 6.4e−15; Figure 3D). Notably, M103 was significantly overexpressed in MSS versus MSI-H tumors (Adj*p* = 3.6e−08, Figure S2). This divergence suggests that MSI-H tumors may feature immune-mediated tumor control, whereas MSS tumors may adopt metabolic adaptations that support immune evasion and tumor progression.

### Diversity and clonal composition of T cells in dMMR CRC tumors

**Figure 4:**
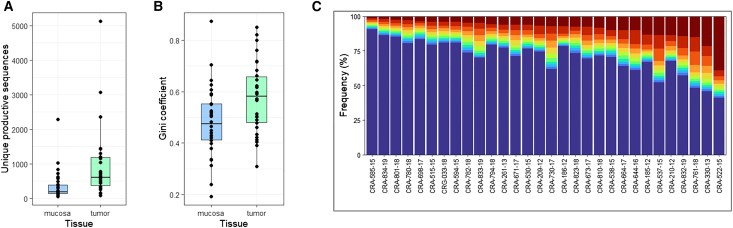
Clonality of TRB repertoires in tumor and histologically normal mucosa (A) Comparison of the number of unique TRB sequences that are in-frame and free of premature stop codons. Tumor tissue contained a significantly higher number of unique productive TRB-CDR3 sequences than histologically normal mucosa (*p* = 2.9 × 10⁻³). (B) Evaluation of clonal evenness among tissues using the Gini coefficient, with values ranging from 0 (equal abundance) to 1 (completely unequal). Tumors showed significantly higher TRB-CDR3 repertoire inequality than histologically normal mucosa (*p* = 5.2 × 10⁻³), consistent with greater clonal diversity. (C) Cumulative frequency distributions of the 10 most abundant TRB-CDR3 sequences in 30 tumors. Each top sequence is shown in a different color, with progressively cooler hues. All less frequent clones are shown in dark blue. Data are represented as medians with interquartile ranges; whiskers indicate minimum and maximum values.

To understand the functional implications of immune infiltration, we next examined the diversity and clonal composition of lymphocytes infiltrating dMMR CRC tumors in the SOTON cohort using deep targeted sequencing via iRepertoire. For T cells, our analysis focused on the nucleotide and amino acid sequences of the third complementarity-determining region (CDR3) of the TCR β chain (TRB), which accounts for most of the variation within the repertoire of αβ-T cells.^26^ Compared with histologically normal mucosa, tumor tissue had a higher number of unique TRB-CDR3 amino acid sequences (*p* = 2.9e−03, Figure 4A). Repertoire inequality, as quantified by the Gini coefficient (where higher values indicate greater clonal dominance and lower values reflect a more diverse, polyclonal repertoire), was significantly elevated in tumors (*p* = 5.2e−03, Figure 4B), suggesting expanded TRB clones. Further assessment of intra-tumoral TRB-CDR3 sequence frequencies showed that two-thirds of patients harbored a single dominant clone, with frequencies ranging from 5.9% to 39.1% (average 11.5% ± 7.7%, Figure 4C).

To determine whether the expanded clones were bystander T cells specific for epitopes unrelated to malignancy, TRB-CDR3 amino acid sequences were compared with previously annotated TRB sequences curated in the VDJdb database.^27^ Of the 355 expanded TRB clonotypes (defined as unique TRB-CDR3 amino acid sequences with a frequency >1% in the tumor repertoire), 13 (3.7%) matched previously published TRB-CDR3s with experimentally verified epitope specificities for influenza A, CMV, EBV, or SARS-CoV-2 (Table S3). Among the remaining intra-tumoral CDR3s, 277 (79.5%) were patient-unique and not detected in the normal mucosa, with clonal frequencies ranging from 1.0% to 39.1% (mean, 2.6% ± 3.1%), suggesting tumor specificity.

Correlation analysis between the TRB Gini coefficient and histopathology T cell scores revealed only non-significant trends (Table S4). CD4^+^ T cells, both in the tumor core and at the tumor edge, as well as Foxp3^+^ T cells across both regions, showed weak or negligible associations with TRB clonal diversity, suggesting limited or complex involvement. Although CD8^+^ T cells at the tumor edge showed a modest positive trend, it did not reach statistical significance (ρ = 0.35, *p* value = 0.06). Collectively, these findings indicate that most T cell subsets do not show a strong linear relationship with TRB clonal dominance in dMMR CRC tumors, suggesting that TRB clonal diversity provides additional insights into tumor heterogeneity beyond what is captured by T cell subset analysis.

To integrate bulk TCR repertoire sequencing with tumor transcriptome profiles, we stratified tumors into “high” and “low” TRB-CDR3 Gini index groups using Hartemink discretization and performed differential gene expression analysis. DESeq2^28^ identified 378 differentially expressed genes (DEGs) at 5% FDR (Table S5). Gene Ontology (GO) analysis showed that the positively correlated genes (*n* = 196) were enriched for the biological process term “Regulation of immune response” (GO:0050776, FDR = 1.3e−15). These included regulators of T cell activation (*CD274*, *ICAM1*, *IL23A*, *LAG3*, *LILRB4*) and IFN-γ response genes (*CCL3*, *CCL4*, *IRF1*, *JAK2*, and *STAT1*). The negatively correlated genes (*n* = 182) were associated with “Oxidative phosphorylation” (GO:0006119, FDR = 3.6e−04) and included multiple components of the mitochondrial inner membrane (*CKMT2*, *CYP2B6*, *MT-ATP6*, *MT-ATP8*, *MT-CO1-CO3*, *MT-CYB*, *MT-ND1-ND3*, an *PDK4*). Additional enriched genes were linked to “Immunoglobulin (Ig) production” (GO:0002377, FDR = 2.2e−04), including the constant regions of IgD, IgG2, and IgG4, as well as multiple V region genes.

To investigate whether transcriptional programs associated with T cell clonal expansion in the SOTON dMMR cohort also relate to ICI responsiveness in MSS tumors, we computed the module membership metric (kME) for each gene in both expression datasets. We then correlated these kME values with the signed, log_10_-transformed *p* values from differential expression analyses, with the sign indicating the direction of the log2-fold change.

**Figure 5:**
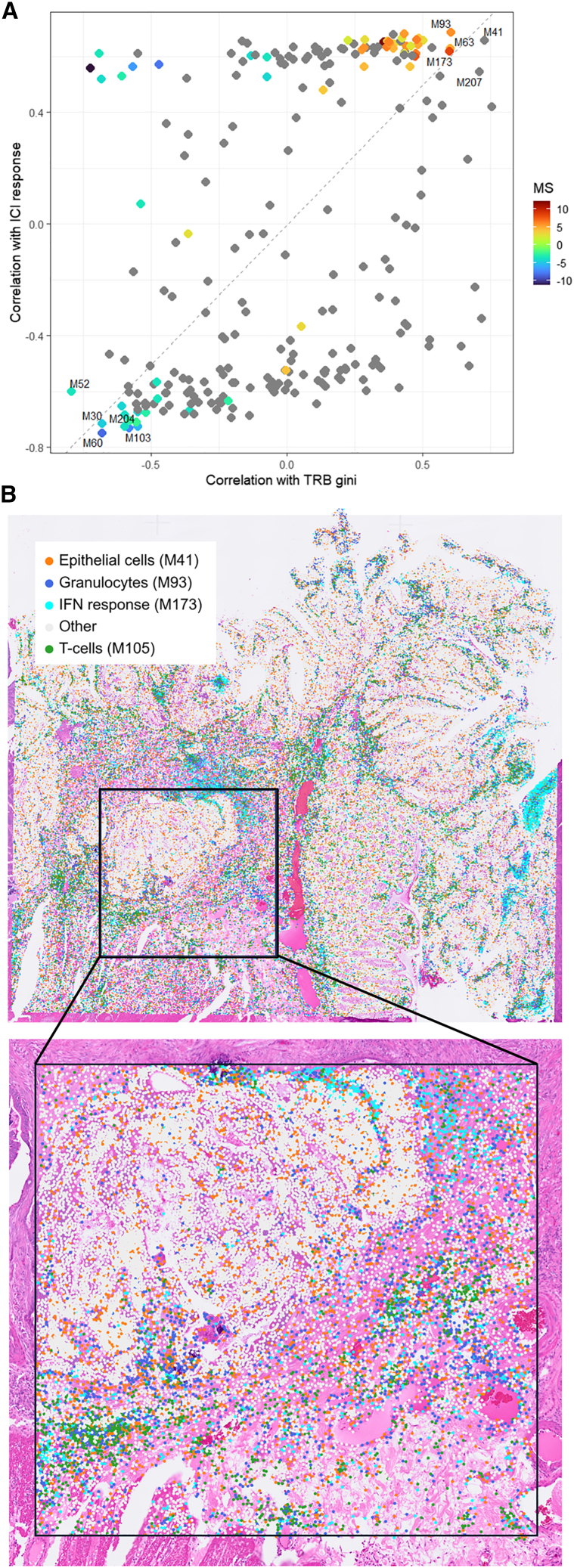
Integrated correlation and spatial mapping of transcriptional modules linking T cell clonality, ICI responsiveness, and immune-epithelial crosstalk in CRC (A) Scatterplot showing the relationship between module-level correlation with T cell clonal expansion (*x* axis, TRB Gini coefficient) and ICI response (*y* axis). Each point represents a co-expression module, colored by the signed log_10_-transformed adjusted *p* value from differential expression (MSI-H vs. MSS), with the sign indicating direction of change. Modules with the strongest combined correlations (top 5 and bottom 5 by summed correlation) are labeled. (B) Spatial transcriptomic mapping of a CRC tissue section at 2-μm resolution, colored by high module activity (>90^th^ percentile) for selected transcriptional programs. The inset highlights a representative region of immune-epithelial interaction, showing colocalization of epithelial and immune-enriched modules. Cell-level annotations were derived by intersecting spatial gene expression with WGCNA-defined module labels and visualized using Scanpy.

Although M173 expression was positively associated with both the TRB Gini coefficient (ρ = 0.60) and ICI responsiveness (ρ = 0.62), the module with the strongest combined correlation was M41 (“MHC class I-like antigen recognition-like”). M41 showed Spearman correlation coefficients of ρ = 0.73 with TRB Gini-associated DEGs and ρ = 0.66 with ICI response-associated DEGs (both *p* < 2.2e−16; Figure 5A). These findings suggest that M41 may represent a shared transcriptional program linking T cell clonal expansion in MSI-H tumors to ICI responsiveness in a subset of MSS tumors. Genes within M41 include regulators of immune modulation (*MICB*, *PROCR*, and *RAET1L*), cell cycle and proliferation (*AKT1*, *CCND1*, *DUS4L*, and *RHEB*), vesicular trafficking and cytoskeletal remodeling (*KLC3*, *PACSIN3*, and *TRIP10*), and epithelial plasticity or barrier function (*CLDN14* and *SLC4A1AP*). Several zinc finger proteins (*ZNF26*, *ZNF416*, *ZNF501*, and *ZNF547*) and long non-coding RNAs (*DDN-AS1* and *LINC01547*) suggest that transcriptional regulation and epigenetic modulation may also contribute to this signature. Notably, M41 expression did not differ significantly between MSI subtypes (Adj*p* = 0.117), indicating its relevance across MSI contexts.

In addition to M41, M93 showed strong correlations with both TRB Gini coefficient-associated DEGs (ρ = 0.61) and ICI response-associated DEGs (ρ = 0.69), suggesting a dual role in immune activation and therapeutic responsiveness. Unlike M41, M93 was significantly overexpressed in MSI-H tumors (Adj*p* = 1.8e−06; Figure S2), underscoring its preferential expression in hypermutated MSI-H tumors. M93 is a component of the immune-related subgraph SG1 (Figure 1A) and is strongly enriched for the Reactome pathway “Immunoregulatory interactions between a lymphoid and a non-lymphoid cell” (NES = 3.14, FDR = 1.2e−26). Genes within M93 include *CCL18*, *CEACAM4*, *EREG*, *IRAK2*, *SLC1A3*, *TNFRSF12A*, and *ZNF267*, many of which are overrepresented in granulocytes. Additional genes, including *CD274* (PD-L1), *SERPINB9*, and *TGM2*, suggest a potential immunosuppressive role. M93 strongly correlated with M105 (CD8^+^ T cell module) in MSI-H tumors (ρ = 0.68), a relationship that was attenuated in MSI-L (ρ = 0.35) and MSS tumors (ρ = 0.56; Figure 2F), indicating context-dependent immune interactions.

To validate the spatial localization of M41- and M93-associated transcriptional programs, we integrated bulk RNA-seq findings with high-definition spatial profiling of CRC.^29^ Although only 13 of the 47 M41 genes were present in the spatial dataset, we developed a Scanpy-based pipeline to overlay the average expression of M41 (13/47), M93 (28/43), M105 (89/96), and M173 (43/56) modules onto spatial coordinates. Cells were annotated based on high expression of module-specific genes, revealing colocalization of M41-expressing epithelial cells with IFN-response, granulocyte, and CD8 T-cell-enriched regions (Figure 5B). This spatial pattern mirrored the correlation structure observed in bulk RNA-seq, reinforcing the biological relevance of M41 and demonstrating the utility of spatial transcriptomics for contextualizing bulk-derived gene modules within the TME.

Together, the spatial and transcriptomic features of M41 suggest that this module captures a transcriptional axis of immune-epithelial crosstalk, characterized by coordinated immune engagement, epithelial remodeling, and metabolic adaptation. The integration of immune-related and tumor-intrinsic genes within M41 underscores the complex interplay between T cell-driven immune pressure and tumor cell plasticity, which may shape the outcome of ICI therapy. In this context, a kME-ranked gene list for M41 in the ICI response dataset showed strong enrichment for the Reactome pathway “Immunoregulatory interactions between a lymphoid and a non-lymphoid cell” (NES = 3.36, Adj*p* = 1.8e−25). Leading-edge genes contributing to this enrichment included a broad array of immune checkpoint molecules, adhesion receptors, and MHC-related genes, such as *CD3D*, *CD3E*, *CD8A*, *CD40*, *CD40LG*, *ICAM1-3*, *HLA-B*/*C*/*E*/*F*, *MICB*, *LILRB1-5*, *SIGLEC1*/*5*/*6*/*7*/*9*/*10*, and *TYROBP*. This reinforces the notion that M41 captures a transcriptional axis of immune-epithelial crosstalk, potentially critical for orchestrating effective anti-tumor immunity.

In summary, M41 and M93 together integrate signals from immune activation, metabolic reprogramming, and tumor structural remodeling. While M41 appears broadly relevant across MSI contexts, M93 may represent a hypermutation-specific immune regulatory axis with potential immunosuppressive features. These modules highlight distinct yet complementary transcriptional programs that link T cell clonality and tumor-intrinsic adaptation, offering potential biomarkers of ICI responsiveness and mechanistic insights into immune-tumor interactions.

### Diversity and clonal composition of B cells in dMMR CRC tumors

Having characterized the T cell repertoire and its transcriptional correlates, we next examined B cell clonal architecture to assess its contribution to anti-tumor immunity in dMMR CRC. Notably, a polyclonal TRB repertoire correlated significantly with three modules: M181 (ρ = 0.56, *p* = 1.2e−03), M186 (ρ = 0.56, *p* = 2.0e−03), and M130 (ρ = 0.55, *p* = 1.2e−02). Two of these (M181 and M186) were enriched for B cell differentiation states. Specifically, M186 was enriched (Adj*p* = 3.8e−03) for class-switched memory B cells (*BANK1*, *IGHA2*, *PAX5*, and *SPIB*), whereas M181 showed highly significant enrichment (Adj*p* = 3.6e−30) for plasma cells (*FCRLA*, *HERPUD*1, *JCHAIN*, *MZB1*, *TNFRSF17*, and multiple V region genes).

**Figure 6:**
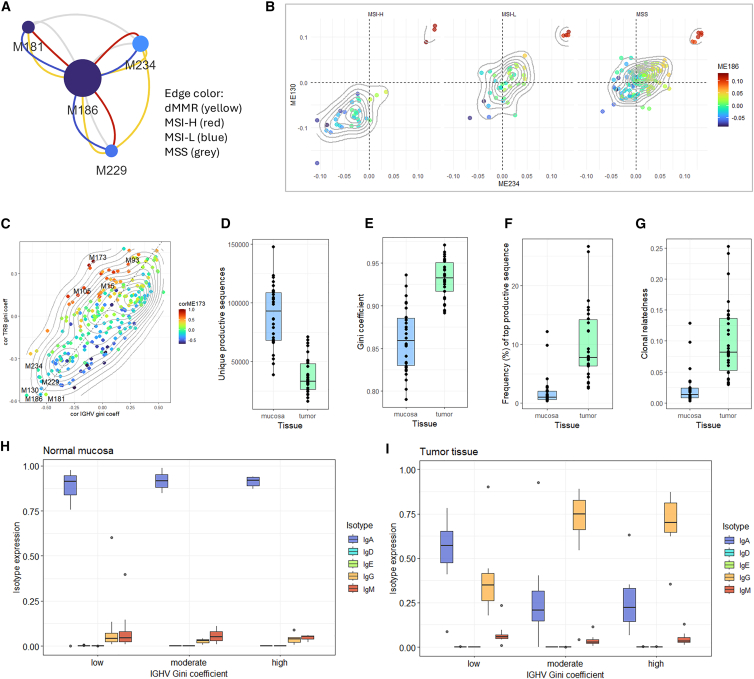
Clonality and isotype distribution of IgH repertoires in tumor and histologically normal mucosa (A) Results of the RWR algorithm with M186 as the seed node, with the top three k-ranked neighbors shown. Edge threshold ρ > 0.87. Nodes are colored according to the correlation between ME expression and the TRB-CDR3 Gini coefficient. (B) Scatterplot of M234 (MADCAM1^+^) versus M130 module expression, split by MSI status, with points colored according to M186 (memory B cell) expression. (C) Scatterplot of IGH-CDR3 versus TRB-CDR3 Gini coefficients, with points colored according to M173 (IFN-γ response) expression. (D) Comparison of the number of unique IGHV sequences that are in-frame and do not contain a premature stop codon. Tumor tissue contained significantly fewer unique productive IGHV-CDR3 sequences than histologically normal mucosa (*p* = 2.0 × 10⁻⁶). (E) Comparison of clonal evenness among tissues, as evaluated by the Gini coefficient, with values ranging from 0 (equal abundance) to 1 (completely unequal). Tumors showed significantly greater IGHV-CDR3 clonotype-size inequality than histologically normal mucosa (*p* = 2.5 × 10⁻⁶). (F) Frequency distributions of the top productive IGHV-CDR3 sequences. The frequencies of the most abundant IGHV-CDR3 sequences were significantly higher in tumors than in histologically normal mucosa (*p* = 1.2 × 10⁻⁵). (G) Comparison of clonal relatedness, defined as the total number of distinct unique sequences with an edit distance <10 from the most frequent sequence, divided by the total number of unique sequences. Values range from 0 (no sequences are related) to 1 (all sequences are related). Clonal relatedness was significantly higher in tumors than in histologically normal mucosa (*p* = 1.3 × 10⁻⁵). (H and I) Isotype distribution across tissues and clonal evenness scores, with the IGHV Gini coefficient converted into 3 discrete values. Data are represented as medians with interquartile ranges; whiskers indicate minimum and maximum values.

Exploration of modules within three edges of M186 in the EGN revealed associations with the vascular endothelial cell modules M229 and M234, independent of MSI status (Figure 6A). Among the genes in M234 is *MADCAM1*, which supports lymphocyte recruitment into the gut mucosa via interactions with ITGA4/ITGB7 or L-selectin.^30^ Compared with MSI-H CRC, M130 is markedly overexpressed in MSS and MSI-L tumors (Adj*p* = 5.3e−12 and Adj*p* = 4.7e−08, respectively; Figure S2). Interestingly, a small number of samples show high expression of both M130 and M186, regardless of MSI status (Figure 6B), which we speculate indicates the presence of tertiary lymphoid structures (TLS). In this context, M130 is enriched for genes that support a B cell niche, including *C7*, *GFRA1*, *ID3*, *PLAG1*, and *WNT5B*.^31^

We observed that the IGHV-CDR3 and TRB-CDR3 Gini coefficients were positively correlated with module expression (Figure 6C). We also found negative correlations between IGHV-CDR3 and M186 (ρ = −0.67, *p* value = 6.6e−05), M130 (ρ = −0.55, *p* value = 1.9e−03), M229 (ρ = −0.56, *p* value = 1.7e−03), and M234 (ρ = −0.57, *p* value = 1.2e−03). These findings support the premise that the vascular endothelial gene signatures of modules M229 and M234 facilitate the recruitment of polyclonal memory B and T cells.

Compared with histologically normal mucosa, tumor tissue showed fewer unique IGHV-CDR3 amino acid sequences (*p* = 2.0e−06, Figure 6D) and greater inequality in clonotype size (*p* = 2.5e−06, Figure 6E). The frequencies of the most abundant IGHV-CDR3 sequences (Figure 6F) and their clonal relatedness (Figure 6G) were also significantly higher (*p* = 1.2e−05 and *p* = 1.3e−05, respectively), consistent with antigen-driven clonal B cell expansion in the tumor. We next examined the distribution of relative abundances of each IGHV-CDR3 sequence and Ig isotype usage. In normal mucosa, IgA predominance over other Ig isotypes remained unchanged across increasing Gini coefficients for the heavy chain (Figure 6H). In contrast, in the tumor, IgA dominance shifted to IgG, accompanied by an increase in the Gini index (Figure 6I), consistent with a T-helper 1-driven B cell response. Together, these findings suggest that B cell responses in dMMR CRC reflect a dual dynamic: antigen-driven clonal expansion of tumor-reactive B cells, as evidenced by increased IGHV clonality and IgG switching, alongside recruitment of diverse, polyclonal B cells supported by vascular and stromal niches. This interplay, potentially orchestrated within TLS, may enable both targeted immune responses and sustained immune surveillance.

## Discussion

In this study, we used a systems-level integrative approach to map the transcriptional and immune landscape of dMMR CRC. By integrating bulk RNA-seq, immune repertoire profiling, and spatial transcriptomics, we identified co-expression modules that capture key features of the TME, including immune activation, metabolic rewiring, and epithelial remodeling. These modules were consistently preserved across independent datasets and reliably distinguished tumors by MSI status.

Among these, module M173 emerged as a robust marker of MSI-high tumors, characterized by elevated expression of IFN-γ response genes and a strong correlation with CD8^+^ T cell activity (module M105). Notably, this immune axis was also detectable in a distinct subset of MSS tumors, challenging the conventional view that immunologically active TMEs are exclusive to MSI-H CRC. This finding suggests that a meaningful proportion of MSS CRCs may harbor features of immune responsiveness. Consistent with our observations, Acha-Sagredo et al. recently described a subset of MSS colorectal tumors exhibiting a constitutive IFN-high immunophenotype.^25^

In addition, modules M41 and M93, which include genes involved in immune checkpoint signaling, antigen presentation, and epithelial plasticity, were enriched in MSS patients who were partial responders to ICI therapy. This points to a previously underappreciated immunologically active phenotype in a subgroup of MSS CRCs. Together, these results highlight that transcriptional programs reflecting T cell clonality and immune-epithelial interactions could serve as biomarkers to identify MSS patients who may still benefit from ICIs despite being classified as ineligible based on MSI status alone. This has significant clinical implications. Currently, only dMMR or MSI-H CRCs are routinely treated with ICIs, leaving the majority (∼85%) of CRC patients with pMMR or MSS tumors without immunotherapy options. Our data support reconsidering this strategy by demonstrating that, in some instances, MSS tumors exhibit immune-active transcriptional signatures akin to those seen in dMMR CRCs. These results imply that patient selection for ICI treatment could be improved by focusing on MSS tumors with immune-active transcriptional profiles, rather than solely relying on MSI testing. This biomarker-based approach could expand the number of patients who benefit from immunotherapy while still using the existing MSI-based guidelines. Accordingly, a prospective, biomarker-guided clinical trial that enriches for immune-active MSS tumors is needed to confirm efficacy and clarify how to incorporate this approach into existing MSI-based care pathways.

We uncovered a complementary epithelial axis with implications for immune escape and therapy resistance. The epithelial module M103, enriched for OXPHOS and fatty-acid metabolism, was elevated in MSS tumors and in progressive disease in the external ICI dataset. In contrast, partial responders showed enrichment of T cell and stromal/myofibroblast programs. These findings support a model in which tumor-intrinsic metabolic states, particularly high OXPHOS and coupled lipid metabolism, oppose effective antitumor immunity. Mechanistically, high tumor OXPHOS intensifies hypoxia, amplifying CD39/CD73-adenosine immunosuppression, while persistent glycolytic flux acidifies the milieu; both changes blunt CD8^+^-T cell recruitment and cytotoxicity.^32^^,^^33^ Heightened mitochondrial activity also increases redox stress, undermining TCR signaling and T cell fitness.^34^ In parallel, lipid-reprogrammed niches accumulate cholesterol and long-chain fatty acids.^35^ Cholesterol induces an exhausted, PD-1^+^ state via ER stress/XBP1, and CD36-dependent fatty acid uptake triggers lipid peroxidation/ferroptosis in CD8^+^ T cells, thereby reducing effector numbers.^35^^,^^36^ The inverse behavior of immune-high (M173/M105) and OXPHOS-high (M103) programs across discovery and validation cohorts is thus consistent with a coherent immune-metabolic axis that shapes ICI outcomes. Translationally, these data support the use of biomarker-guided metabolic ICI combinations in MSS CRC.

Beyond T cells, our data reveal clonally expanded B cell populations in dMMR tumors, with evidence of class switching from IgA to IgG. Modules enriched for memory B cells and plasma cells are associated with vascular endothelial gene signatures, suggesting the presence of TLS that could sustain local immune responses and may represent an additional layer of ICI responsiveness.

Recent studies highlight the rapid growth of multi-omics in drug discovery, with a focus on personalized medicine, AI-driven integration, and resistance mechanisms.^37^^,^^38^ In cancer research, integrating network analysis with single-cell and spatial multi-omics is increasingly common to identify targets, improve biomarker localization, and strengthen external validation. Our approach, using bulk RNA-seq for module identification, immune repertoire profiling for clonal structure, and spatial transcriptomics for cellular context provides an integrated framework for robust, complementary validation of module-level biomarkers. These biomarkers are latent network entities that single-gene tests do not adequately capture.

Collectively, our findings underscore the importance of considering immune-epithelial crosstalk, T cell clonality, and the metabolic landscape when predicting ICI benefit in CRC. By integrating transcriptional and spatial data, our study provides a framework for refining patient selection beyond MSI status alone. It supports the design of biomarker-driven trials to extend immunotherapy to carefully selected MSS patients.

### Limitations of the study

This study focused on system-level network discovery rather than validating individual gene biomarkers. Our single-center cohort (*n* = 30) exceeds the recommended minimum for WGCNA (≥15–20 samples), supporting stable module detection. However, hub gene inference with small sample sizes remains uncertain. Therefore, we emphasized cross-cohort module preservation in TCGA-CRC, where 84.9% of SOTON modules were preserved. We also replicated expected MSI-related differences between immune-high and OXPHOS-high programs, suggesting that the findings are likely applicable beyond our center.

To address heterogeneity more fully, single-gene confirmation is intentionally deferred. Because co-expression modules are latent constructs that summarize multi-gene programs across diverse cell types, per-gene assays (e.g., qPCR) cannot, by themselves, validate the network components. Accepted orthogonal validations for module-level biomarkers include module preservation across independent cohorts and consistent association with clinical response in external datasets,^39^^,^^40^ the approaches we implemented here. In line with recent perspectives on tumor immune microenvironment heterogeneity and biomarker development, future multicenter studies (≥200 CRC patients) will be required to power gene-level assays and outcome analyses at scale.^41^

In addition to cohort size, two technical limitations are worth noting. First, although immune repertoire sequencing identified clonally expanded T and B cell populations, we did not functionally verify the antigen specificity or effector functions of these clones. Second, although spatial transcriptomics localized key transcriptional modules, the limited gene coverage in the spatial dataset constrained our ability to characterize the spatial dynamics of immune-epithelial interactions fully.

## Resource availability

### Lead contact

Requests for further information and resources should be directed to the lead contact, Stephen M. Thirdborough (smt1@soton.ac.uk).

### Materials availability

This study did not generate unique reagents.

### Data and code availability

• Data: bulk RNA-seq data from the SOTON cohort have been deposited in the NCBI Gene Expression Omnibus (GEO) under accession GSE303444 and are publicly available. Immune repertoire (TCR/BCR) sequencing data are available in the AIRR Data Commons at VDJServer Community Data Portal with UUID: 6d276a73-765e-4c90-9b31-e96e90f48a44.
• Accession numbers are listed in the key resources table.
• Code: this study does not report original code.
• Additional information: any additional information required to reanalyze the data reported in this paper is available from the lead contact upon request.

## Acknowledgments

We gratefully acknowledge funding from 10.13039/100018063Bowel Research UK and Wessex Medical Research, which enabled this study. We thank the University of Southampton Faculty of Medicine Tissue Bank for managing sample transportation, processing, storage, and release for assay. We also thank the Oxford Genomics Center at the Wellcome Center for Human Genetics (funded by 10.13039/100010269Wellcome Trust grant reference 203141/Z/16/Z) for generating the RNA-seq data, and iRepertoire for subsidizing part of the cost of TCR-sequencing. None of these organizations was involved in the study design or analysis. Finally, we thank Agenus for providing access to transcriptomic data from MSS patients treated with anti-PD-1 and anti-CTLA-4.

## Author contributions

S.L.L., A.S., and S.M.T. contributed to the conception and design of the study. S.L.L., A.C.B., M.A.-K., J.N.P., A.M., and S.M.T. contributed to the acquisition, analysis, or interpretation of data. S.L.L., A.S., and S.M.T. contributed to drafting the manuscript and preparation of figures.

## Declaration of interests

The authors declare no competing interests.

## STAR★Methods

### Key resources table

**Table:** 

REAGENT or RESOURCE	SOURCE	IDENTIFIER
**Deposited data**		

*SOTON-dMMR bulk RNA-seq*	NCBI-GEO	GSE303444
*BCR and TCR immune repertoire sequencing*	VDJServer Community Data Portal	https://vdjserver.org/community 6d276a73-765e-4c90-9b31-e96e90f48a44
TCGA-CRC	GDC Data Portal	https://portal.gdc.cancer.gov/
Spatial transcriptomics	10*x* Genomics	https://www.10xgenomics.com/platforms/visium/product-family/dataset-human-crc

**Software and algorithms**		

STAR 2.7.11b	Dobin et al.^42^	https://github.com/alexdobin/STAR/releases
Python 3.11.5	Python Software Foundation	https://python.org
Bin2cell 3.0.0	Polanski et al.^43^	https://github.com/Teichlab/bin2cell
Stardist 0.9.1	Schmidt et al.^44^	https://github.com/stardist/
Scanpy 1.10.3	Wolf et al.^45^	https://github.com/scverse/scanpy
R v4.4.1	CRAN/Bioconductor	https://cran.r-project.org/bin/windows/base/
Rsubread v2.20.0	Bioconductor	https://bioconductor.org/packages/
edgeR v4.2.1	Bioconductor	https://bioconductor.org/packages/
DESeq2 v1.44.0	Bioconductor	https://github.com/thelovelab/DESeq2
LymphoSeq v1.32.0	Bioconductor	https://github.com/davidcoffey/LymphoSeq
WGCNA v1.73	CRAN/Bioconductor	https://cran.r-project.org/bin/windows/base/
Survminer v0.5.0	CRAN	https://cran.r-project.org/bin/windows/base/
GeneOverlap 1.40.0	Bioconductor	https://github.com/shenlab-sinai/geneoverlap
fgsea v1.30.0	Bioconductor	https://github.com/alserglab/fgsea

### Experimental model and study participant details

#### Human subjects

Tumor and adjacent normal colorectal tissue were collected from 30 treatment-naïve patients with dMMR CRC (SOTON cohort) at University Hospital Southampton. Ethical approval was obtained from the University of Southampton Research Ethics Committee (07/H0504/125). All patients provided informed consent. The cohort consisted of 20 female and 10 male patients with a median age of 77 years (range 53–89). Sex was recorded from clinical records; the study was not powered to test sex-specific associations with the reported transcriptomic or repertoire outcomes, and no sex-stratified analyses were performed. Information regarding participants’ race and ethnicity was not collected.

### Method details

#### MMR status and histopathology

MMR status was determined by immunohistochemistry for MLH1, PMS2, MSH2, and MSH6. Tumors with loss of any MMR protein were classified as dMMR. Clinical staging followed the Union for International Cancer Control tumor-node-metastasis criteria.

#### Bulk RNA-sequencing

RNA was extracted from snap-frozen tumor sections (14–22 μm) using the RNeasy Plus Mini Kit. Libraries were prepared using NEBNext kits and sequenced on an Illumina NovaSeq6000 (150 bp paired end). Reads were aligned to GRCh38.114 using STAR,^42^ quantified with featureCounts, and normalized using edgeR (v4.2.1).

#### Immune repertoire sequencing

Total RNA was submitted to iRepertoire for TCR and BCR sequencing. Libraries were generated using multiplex primers with UMIs and sequenced on an Illumina NextSeq. TRB-CDR3 and IGH-CDR3 sequences were analyzed using LymphoSeq (v1.32.0) and annotated with VDJdb.^27^

#### Differential gene expression and enrichment analysis

Differentially expressed genes were identified using DESeq2 (v1.44.0) with FDR ≤0.05. Log-fold changes were modeled using apeglm. Gene set enrichment was performed using fgsea (v1.30.0) with MSigDB and Reactome pathways.

#### Weighted gene co-expression network analysis

WGCNA was performed on log-CPM-transformed data using Spearman correlations. Modules were defined using dynamic tree cutting. Module preservation in TCGA-CRC was assessed with 200 permutations. Cell type enrichment was inferred using EnrichR.

#### Spatial transcriptomics

Public Visium HD CRC data were processed using bin2cell.^43^ Cell segmentation was performed with StarDist^44^ and label propagation. Expression matrices were filtered and normalized using Scanpy. Module activity was mapped by intersecting spatial gene sets with the SOTON WGCNA modules. Cells were annotated based on high module expression (>90th percentile).

#### Survival analysis

Univariate Cox proportional hazards models were fitted using the R survival package. Optimal cutoffs for ME expression were determined using surv_cutpoint from the R package survminer.

### Quantification and statistical analysis

All statistical analyses were performed in R (v4.4.1). Group comparisons used Kruskal-Wallis tests. Multiple testing correction was applied using the BH method. No data imputation was performed.

## Supplemental information

Document S1. Figures S1 and S2

Table S1. Clinical and histopathological metadata for the SOTON dMMR CRC cohort

Table S2. Annotations and enrichment results are provided for all 239 modules identified in the SOTON cohort

Table S3. Expanded TRB-CDR3 clonotypes and matches to VDJdb annotated specificities

Table S4. Correlations between TRB repertoire clonality (Gini index) and histopathology-derived immune cell scores

Table S5. Differentially expressed genes between high and low TRB-CDR3 Gini index tumor groups

## References

[1] Araghi M., Arnold M., Rutherford M.J., Guren M.G., Cabasag C.J., Bardot A., Ferlay J., Tervonen H., Shack L., Woods R.R. (2021). Colon and rectal cancer survival in seven high-income countries 2010-2014: variation by age and stage at diagnosis (the ICBP SURVMARK-2 project). Gut.

[2] Guinney J., Dienstmann R., Wang X., de Reyniès A., Schlicker A., Soneson C., Marisa L., Roepman P., Nyamundanda G., Angelino P. (2015). The consensus molecular subtypes of colorectal cancer. Nat. Med..

[3] Samowitz W.S., Curtin K., Ma K.N., Schaffer D., Coleman L.W., Leppert M., Slattery M.L. (2001). Microsatellite instability in sporadic colon cancer is associated with an improved prognosis at the population level. Cancer Epidemiol. Biomarkers Prev..

[4] Sinicrope F.A., Sargent D.J. (2012). Molecular pathways: microsatellite instability in colorectal cancer: prognostic, predictive, and therapeutic implications. Clin. Cancer Res..

[5] Boland C.R., Goel A. (2010). Microsatellite instability in colorectal cancer. Gastroenterology.

[6] Tutlewska K., Lubinski J., Kurzawski G. (2013). Germline deletions in the EPCAM gene as a cause of Lynch syndrome - literature review. Hered. Cancer Clin. Pract..

[7] Mlecnik B., Bindea G., Angell H.K., Maby P., Angelova M., Tougeron D., Church S.E., Lafontaine L., Fischer M., Fredriksen T. (2016). Integrative Analyses of Colorectal Cancer Show Immunoscore Is a Stronger Predictor of Patient Survival Than Microsatellite Instability. Immunity.

[8] Llosa N.J., Cruise M., Tam A., Wicks E.C., Hechenbleikner E.M., Taube J.M., Blosser R.L., Fan H., Wang H., Luber B.S. (2015). The vigorous immune microenvironment of microsatellite instable colon cancer is balanced by multiple counter-inhibitory checkpoints. Cancer Discov..

[9] Morris V.K., Kennedy E.B., Baxter N.N., Benson A.B., Cercek A., Cho M., Ciombor K.K., Cremolini C., Davis A., Deming D.A. (2023). Treatment of Metastatic Colorectal Cancer: ASCO Guideline. J. Clin. Oncol..

[10] Chalabi M., Fanchi L.F., Dijkstra K.K., Van den Berg J.G., Aalbers A.G., Sikorska K., Lopez-Yurda M., Grootscholten C., Beets G.L., Snaebjornsson P. (2020). Neoadjuvant immunotherapy leads to pathological responses in MMR-proficient and MMR-deficient early-stage colon cancers. Nat. Med..

[11] Chalabi M., Verschoor Y.L., Tan P.B., Balduzzi S., Van Lent A.U., Grootscholten C., Dokter S., Büller N.V., Grotenhuis B.A., Kuhlmann K. (2024). Neoadjuvant Immunotherapy in Locally Advanced Mismatch Repair-Deficient Colon Cancer. N. Engl. J. Med..

[12] Cercek A., Lumish M., Sinopoli J., Weiss J., Shia J., Lamendola-Essel M., El Dika I.H., Segal N., Shcherba M., Sugarman R. (2022). PD-1 Blockade in Mismatch Repair-Deficient, Locally Advanced Rectal Cancer. N. Engl. J. Med..

[13] Cercek A., Sinopoli J.C., Shia J., Weiss J.A., Temple L., Smith J.J., Saltz L.B., Widmar M., Fumo G., Aparo S. (2024). Durable complete responses to PD-1 blockade alone in mismatch repair deficient locally advanced rectal cancer. J. Clin. Oncol..

[14] Kawakami H., Zaanan A., Sinicrope F.A. (2015). Microsatellite instability testing and its role in the management of colorectal cancer. Curr. Treat. Options Oncol..

[15] Le D.T., Uram J.N., Wang H., Bartlett B.R., Kemberling H., Eyring A.D., Skora A.D., Luber B.S., Azad N.S., Laheru D. (2015). PD-1 Blockade in Tumors with Mismatch-Repair Deficiency. N. Engl. J. Med..

[16] Chen E.X., Jonker D.J., Loree J.M., Kennecke H.F., Berry S.R., Couture F., Ahmad C.E., Goffin J.R., Kavan P., Harb M. (2020). Effect of Combined Immune Checkpoint Inhibition vs Best Supportive Care Alone in Patients With Advanced Colorectal Cancer: The Canadian Cancer Trials Group CO.26 Study. JAMA Oncol..

[17] Vaccaro G.M., Rothe M., Mangat P.K., Garrett-Mayer E., Hwang J.J., Alese O.B., Khalil M.F., Hameed M.K., Duvivier H.L., Cannon T.L. (2022). Nivolumab plus ipilimumab (N+I) in patients (pts) with colorectal cancer (CRC) with high tumor mutational burden (hTMB): Results from the Targeted Agent and Profiling Utilization Registry (TAPUR) study. J. Clin. Oncol..

[18] Chand D., Savitsky D.A., Krishnan S., Mednick G., Delepine C., Garcia-Broncano P., Soh K.T., Wu W., Wilkens M.K., Udartseva O. (2024). Botensilimab, an Fc-Enhanced Anti-CTLA-4 Antibody, Is Effective against Tumors Poorly Responsive to Conventional Immunotherapy. Cancer Discov..

[19] Bullock A.J., Schlechter B.L., Fakih M.G., Tsimberidou A.M., Grossman J.E., Gordon M.S., Wilky B.A., Pimentel A., Mahadevan D., Balmanoukian A.S. (2024). Botensilimab plus balstilimab in relapsed/refractory microsatellite stable metastatic colorectal cancer: a phase 1 trial. Nat. Med..

[20] Avallone A., De Stefano A., Pace U., Catteau A., Di Gennaro E., Tatangelo F., Boquet I., Cassata A., Costantini S., De Franciscis S. (2020). 491P Neoadjuvant nivolumab in early stage colorectal cancer. Ann. Oncol..

[21] Baran B., Mert Ozupek N., Yerli Tetik N., Acar E., Bekcioglu O., Baskin Y. (2018). Difference Between Left-Sided and Right-Sided Colorectal Cancer: A Focused Review of Literature. Gastroenterol. Res..

[22] Langfelder P., Horvath S. (2007). Eigengene networks for studying the relationships between co-expression modules. BMC Syst. Biol..

[23] Cancer Genome Atlas Network (2012). Comprehensive molecular characterization of human colon and rectal cancer. Nature.

[24] Valdeolivas A., Tichit L., Navarro C., Perrin S., Odelin G., Levy N., Cau P., Remy E., Baudot A. (2019). Random walk with restart on multiplex and heterogeneous biological networks. Bioinformatics.

[25] Acha-Sagredo A., Andrei P., Clayton K., Taggart E., Antoniotti C., Woodman C.A., Afrache H., Fourny C., Armero M., Moinudeen H.K. (2025). A constitutive interferon-high immunophenotype defines response to immunotherapy in colorectal cancer. Cancer Cell.

[26] Nikolich-Zugich J., Slifka M.K., Messaoudi I. (2004). The many important facets of T-cell repertoire diversity. Nat. Rev. Immunol..

[27] Shugay M., Bagaev D.V., Zvyagin I.V., Vroomans R.M., Crawford J.C., Dolton G., Komech E.A., Sycheva A.L., Koneva A.E., Egorov E.S. (2018). VDJdb: a curated database of T-cell receptor sequences with known antigen specificity. Nucleic Acids Res..

[28] Love M.I., Huber W., Anders S. (2014). Moderated estimation of fold change and dispersion for RNA-seq data with DESeq2. Genome Biol..

[29] Oliveira M.F.d., Romero J.P., Chung M., Williams S.R., Gottscho A.D., Gupta A., Pilipauskas S.E., Mohabbat S., Raman N., Sukovich D.J. (2025). High-definition spatial transcriptomic profiling of immune cell populations in colorectal cancer. Nat. Genet..

[30] Garrood T., Lee L., Pitzalis C. (2006). Molecular mechanisms of cell recruitment to inflammatory sites: general and tissue-specific pathways. Rheumatology (Oxford).

[31] Huber C., Thielen C., Seeger H., Schwarz P., Montrasio F., Wilson M.R., Heinen E., Fu Y.X., Miele G., Aguzzi A. (2005). Lymphotoxin-beta receptor-dependent genes in lymph node and follicular dendritic cell transcriptomes. J. Immunol..

[32] Apostolova P., Pearce E.L. (2022). Lactic acid and lactate: revisiting the physiological roles in the tumor microenvironment. Trends Immunol..

[33] Steingold J.M., Hatfield S.M. (2020). Targeting Hypoxia-A2A Adenosinergic Immunosuppression of Antitumor T Cells During Cancer Immunotherapy. Front. Immunol..

[34] Kuo C.L., Ponneri Babuharisankar A., Lin Y.C., Lien H.W., Lo Y.K., Chou H.Y., Tangeda V., Cheng L.C., Cheng A.N., Lee A.Y.L. (2022). Mitochondrial oxidative stress in the tumor microenvironment and cancer immunoescape: foe or friend?. J. Biomed. Sci..

[35] Ma X., Bi E., Lu Y., Su P., Huang C., Liu L., Wang Q., Yang M., Kalady M.F., Qian J. (2019). Cholesterol Induces CD8(+) T Cell Exhaustion in the Tumor Microenvironment. Cell Metab..

[36] Ma X., Xiao L., Liu L., Ye L., Su P., Bi E., Wang Q., Yang M., Qian J., Yi Q. (2021). CD36-mediated ferroptosis dampens intratumoral CD8(+) T cell effector function and impairs their antitumor ability. Cell Metab..

[37] Liu Y., Zhang S., Liu K., Hu X., Gu X. (2024). Advances in drug discovery based on network pharmacology and omics technology. Curr. Pharmaceut. Anal..

[38] Xu Z., Li W., Dong X., Chen Y., Zhang D., Wang J., Zhou L., He G. (2024). Precision medicine in colorectal cancer: Leveraging multi-omics, spatial omics, and artificial intelligence. Clin. Chim. Acta..

[39] Langfelder P., Luo R., Oldham M.C., Horvath S. (2011). Is my network module preserved and reproducible?. PLoS Comput. Biol..

[40] Langfelder P., Horvath S. (2008). WGCNA: an R package for weighted correlation network analysis. BMC Bioinf..

[41] Keenan B.P., Yadav M., Ansstas G., Fabrizio D., Murugesan K., Montesion M., Guha Niyogi D., Mellman I., Melero I. (2026). Intratumoral heterogeneity and immunotherapy resistance: clinical implications. Ann. Oncol..

[42] Dobin A., Davis C.A., Schlesinger F., Drenkow J., Zaleski C., Jha S., Batut P., Chaisson M., Gingeras T.R. (2013). STAR: ultrafast universal RNA-seq aligner. Bioinformatics.

[43] Polański K., Bartolomé-Casado R., Sarropoulos I., Xu C., England N., Jahnsen F.L., Teichmann S.A., Yayon N. (2024). Bin2cell reconstructs cells from high resolution Visium HD data. Bioinformatics.

[44] Schmidt U., Weigert M., Broaddus C., Myers G., Frangi A.F., Schnabel J.A., Davatzikos C., Alberola-López C., Fichtinger G. (2018). Cell Detection with Star-Convex Polygons. held in Cham.

[45] Wolf F.A., Angerer P., Theis F.J. (2018). SCANPY: large-scale single-cell gene expression data analysis. Genome Biol..

