# Supplementary material for: Immune-epithelial crosstalk governs immune checkpoint inhibitor efficacy in colorectal cancer
Source: iScience. 2026 Jul 8;29(7):116544. doi: 10.1016/j.isci.2026.116544 (PMC13378363; doi:10.1016/j.isci.2026.116544)
Supplement: Document S1. Figures S1 and S2 [file mmc1.pdf]

iScience, Volume *29*

Supplemental information

Immune-epithelial crosstalk governs immune checkpoint inhibitor efficacy in colorectal cancer

Sheah Lin Lee, Adrian C. Bateman, Margaret Ashton-Key, John N. Primrose, Alex Mirnezami, Aymen Al-Shamkhani, and Stephen M. Thirdborough

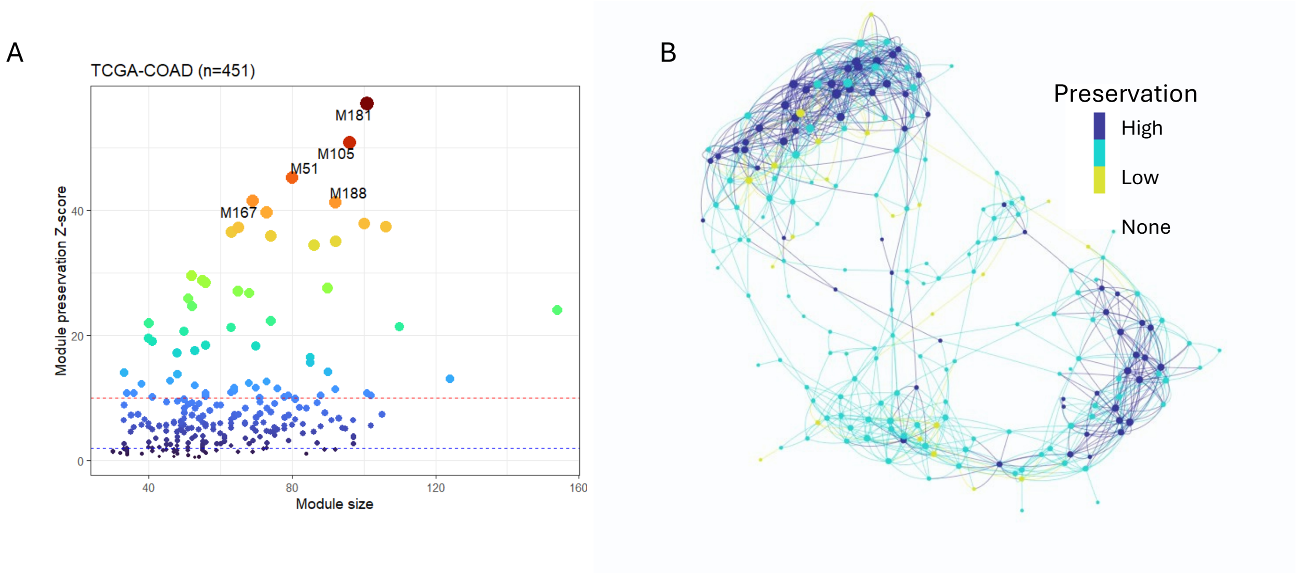

Figure S1 Module preservation analysis in TCGA-COAD test data.

(A) Scatter plot of composite module preservation Z-scores versus module size. The SOTON dMMR CRC expression data were used as reference and TCGA-CRC as the independent test dataset. Values < 2 are not preserved (blue dashed line) while values > 10 (red dashed line) are strongly preserved. (B) Module eigengene network for the SOTON cohort, with the modules according to module preservation Z-scores, where values < 2 (green-yellow) indicate non-preservation, and values > 10 (dark blue) indicate strong preservation.

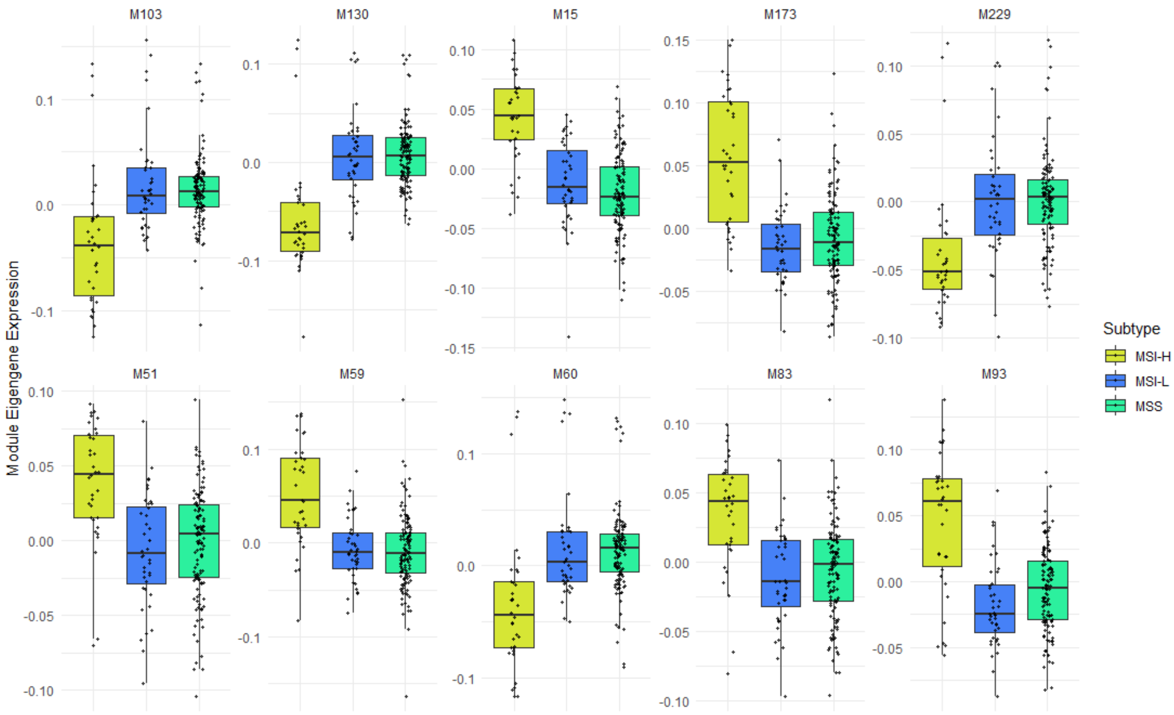

Figure S2. Top 10 modules significantly associated with MSI status.
